# Think regionally, act locally: metals in honeybee workers in the Netherlands (surveillance study 2008)

**DOI:** 10.1007/s10661-016-5451-8

**Published:** 2016-07-12

**Authors:** J. J. M. van der Steen, B. Cornelissen, T. Blacquière, J. E. M. L. Pijnenburg, M. Severijnen

**Affiliations:** 1 Wageningen UR, P.O. Box 16, 6700 AA Wageningen, The Netherlands; 2 Provincie Limburg, Milieu, Onderzoek & Advies, Maastricht, The Netherlands

**Keywords:** Bioindication, Honeybee, *Apis mellifera*, Heavy metals, Surveillance study, Land use, Region

## Abstract

In June 2008, a surveillance study for metals in honeybees was performed in the Netherlands. Randomly, 150 apiaries were selected. In each apiary, five colonies were sampled. Per apiary, the hive samples were pooled. The apiary sample was analysed for Al, As, Ba, Cd, Co, Cr, Cu, Li, Mn, Mo, Ni, Sb, Se, Sn, Sr, Ti, V and Zn. All metals could be detected in all apiaries. As, Li, Sb, Sn and V were detected in part of the apiaries. The overall picture showed a regional pattern. In apiaries in the east of the Netherlands, Al, Ba, Cr, Mn, Mo, Ni, Se and Ti are found in higher concentrations compared to the west. In-region variation was demonstrated, indicating local effects. The vicinity of the apiaries was mapped afterwards and characterised as land uses of >50 % agricultural area, >50 % wooded area, >50 % urban area and mixed land use within a circle of 28 km^2^ around the apiary. The results indicated that in apiaries located in >50 % wooded areas, significantly higher concentrations of Al, Ba, Cd, Cr, Cu, Li, Mn, Mo, Ni, Sb, Sr, Ti and Zn were found compared to agricultural, urban and mixed land use areas. We conclude that (1) the ratio between metal concentrations varies per region, demonstrating spatial differences, and (2) there is in-region local variation per metal. The results indicate the impact of land use on metal concentrations in honeybees. For qualitative bioindication studies, regional, local and land use effects should be taken into account.

## Introduction

Along with collecting nectar, pollen, water and propolis, honeybees pick up particles deposited in the flowers and other places where bees collect resins (propolis) and water. Honeybees also collect the sweet aphid secretion, called honeydew, from the leaves. In addition to pollen collected from the anthers, the branched hairs on the bee’s body easily hold non-floral particles originating from atmospheric deposition. In this way, each honeybee can act as an environment microsampler and a honeybee colony as a sampler unit. In the active foraging period of the honeybee colony, about a quarter of the colony’s population is a forager bee. The number of foragers, actively collecting food, depends on the colony size, the colony’s need for pollen, nectar, water and propolis, the availability of food and the time of year. The number of foraging trips varies from some hundreds to many thousands of trips per day, resulting in hundreds to many thousands microsamples accumulated in the honeybee colony in the hive. The feature of the honeybee of unintentionally collecting non-floral particles makes the honeybee suitable for qualitative bioindication, providing information about the environment.

Metals are a natural component of the bee’s food. In “Honey, a comprehensive survey” by Crane (1979), an overview is presented of metals in honey, showing that dark honeys, often partly made from honeydew, contain higher concentrations of metals compared to light ones. For example, the average Mg in light honey is 19 ppm and in dark honey is 35 ppm. For Cu, the averages are 0.29 and 0.56, respectively. The concentrations range significantly; the lowest Fe concentration presented is 0.70 ppm and the highest is 33.50 ppm, both in dark honey. In a honey study in southeast Anatolia, the mean concentrations of Mg, Cu, Mn, Zn and Co were 33, 1.8, 1.6, 2.7 and 1.0 ppm, respectively (Yılmaz and Yavuz 1999). Latorre et al. (1999) classified honeys based on the metal content data. The mean concentrations of metals in pollen of 20 samples were determined in a study in Spain by Serra Bonvehi and Escolà Jordà (1997) as Fe, 39.2 ppm; Zn, 33.9 ppm; Cu, 8.7 ppm; and Mg, 432.2 ppm. Campos et al. 2008 present the range of metals in dried pollen for K, 4000–20,000 ppm; Mg, 200–3000 ppm; Ca, 200–3000 ppm; P, 800–6000 ppm; Fe, 11–170 ppm; Zn, 30–250 ppm; Cu, 2–16 ppm; Mn, 20–110 ppm in a study of the detailed composition of bee-collected pollen. The sources of metals detected in honeybees are nectar, honeydew, pollen, plus possible atmospheric deposition of metal-containing particles. Part of the metals will be in the bee because of ingestion of food and part on the exterior of the bee as pollen and non-floral particles. Metals are stored in granules in fat cells and midgut cells. The granules are formed during the short period bees consume pollen. Unlike fat cells, due to regeneration of the midgut, these granules can no longer be found in midgut cells in case pollen feeding stops (Raes et al. 1989). Analysing the complete bee, the result is the sum of what is in and on the bee. In the current study, this is referred to as metals in the bee. It is obvious that any analysis of bees on heavy metals results in detecting metals in varying concentration ranges. Therefore, in bioindication studies, only significantly exceeded concentrations of metals in honeybees studied under defined site conditions indicate an extra exposure of bees to heavy metals and may draw attention for further studies. The concentrations of heavy metals show significant temporal and spatial variations (Lambert et al. 2012; Perugini et al. 2011; Ruschioni et al. 2013; Satta et al. 2012; Steen et al. 2012b). Bioindication studies revealed that high heavy metal concentrations can only be detected in live honeybees and not in honey and dead bees (Ruschioni et al. 2013). The mechanism behind the difference in metal concentrations in dead and live bees was not part of this study. The live bees were forager bees and the dead ones died in the hive; this may explain the difference in exposure to heavy metals brought in by the foragers. According to Satta et al. (2012), sampling foragers gives the best results to detect heavy metals in bees. Land use affects metal concentrations in the honeybee. Apiaries in urban and landscapes with hedgerows contained higher concentrations of Pb in honeybees than in those in cultivated and island landscapes (Lambert et al. 2012). Weather conditions also affect the concentrations of heavy metals in bees; dry weather results in higher concentrations (Lambert et al. 2012; Satta et al. 2012).

Most heavy metal bioindication studies with honeybees are focussed on a limited number of metals and performed at defined sites, e.g. near motorways, airports, industrial sites, agricultural areas and landfill sites. The results are compared to control sites (mostly urban sites or natural reserve parks) to demonstrate differences. Perugini et al. (2011) showed elevated Pb concentrations near the Ciampino airport of Rome compared to three nature reserves and moderately polluted urban areas. Forager bees in a post-mining area in Sardinia contain more Cd and Pb than the ones in the control sites 50 km from the post-mining area (Satta et al. 2012).

The current study presented is, to our knowledge, the first national surveillance study conducted on 18 metals. The objective of this study was to collect data of the spatial variation of metal concentrations in honeybee colonies in the Netherlands. Afterwards, land use in the vicinity of the apiaries was mapped to evaluate the impact of land use (urban, rural, agricultural and mixed sites) on the concentrations of metals in honeybees. As sampling was done in June 2008, only spatial differences were studied. The study included the metals Al, As, Ba, Cd, Co, Cr, Cu, Li, Mn, Mo, Ni, Sb, Se, Sn, Sr, Ti, V and Zn. Following the definition of heavy metals being metals having a periodic system element number exceeding Fe (element no. 26), Al, Li, Mn, Ti and V do not meet this definition. Nevertheless, these metals are included in this study, and the term ‘metals’ in this article represents all metals, including the heavy metals.

## Material and methods

### Number of apiaries sampled

Surveillance was set up to detect both the incidence of honeybee diseases and the concentrations of heavy metals in honeybees in the Netherlands. The number of apiaries sampled was based on the probability to detect honeybee diseases at low prevalence. The number of apiaries to be sampled in order to detect bee diseases is calculated with the binomial probability theory equation: $N=\frac{1\mathrm{n}\left(1-D\right)}{1\mathrm{n}\left(1-P\right)}$, where *N* is the sample size, ln the natural logarithm, *D* is the probability (power) of detection and *P* is the minimal proportion of bees carrying the pathogen (Pirk et al. 2013). With a probability (power) of 0.95 and a minimal proportion of 2 % of the apiaries having a bee disease at low prevalence in the Netherlands, 148 apiaries must be sampled to detect at least one infected apiary. In this study, 150 apiaries were sampled.

### Number of colonies/pooled bee samples

Based on the variance of metal concentrations recorded in three hives per apiary in the study of Steen et al. (2012a), minimally, three colonies should be sampled for a reliable mean apiary recording. Sampling was done by trained beekeepers. Per apiary, five colonies were sampled by collecting about 100 bees from the outer brood frame. As the objective of the study was to obtain the incidence of metal in honeybees per apiary and not the difference between metal concentrations in bees of different colonies in an apiary, the samples were pooled, resulting in one apiary sample. The samples were stored during transportation in a cooling box and next stored until analysis at −20 °C.

### Chemical analysis

Chemical analyses were performed by the environmental laboratory of the Province Limburg (Hoofdgroep Milieu and Water Bureau onderzoek en advies). Per pooled apiary sample, 25 bees were picked randomly, weighed, dried for 24 h at 120 °C and destructed by boiling at 170 °C in aqua regia. The resulting liquid was topped up to 50 ml with demi water. After an overnight rest, the clear top liquid was analysed using inductively coupled plasma atomic emission spectroscopy. The resulting signals (in nanograms per millilitre) were converted to nanograms per gram bee with a conversion factor (volume sample/(weight bees × mean percentage dry weight)). This resulted in concentrations expressed as parts per billion per dry weight. The parts per billion per dry weight was subsequently converted to micrograms metal per gram dry matter bee. The overall weight loss of bee samples as a result of the drying process is 68 % (Steen et al. 2012b).

### Land use

Applying GIS software ArcGis 9.2, land use was mapped using the LGN5 database (landgebruik, unit postcode) in a 28-km^2^ area around the apiary (radius, approximately 3 km). The percentages of land use were calculated with the following parameters: Code 7, arable land; Code 8, glass horticulture; Code 9, orchard; Code 11, wooded area; Code 16, water; Code 18, urban area; Code 25, infrastructure; Code 30, nature. All other land uses were combined as ‘mixed use’. The foraging area circling the apiaries was then defined by the dominant land use type, combining the given land use definitions for the following categories: agricultural, wooded, urban and rest/mixed land use. Areas covering ≥50 % of one of these categories were classified as such.

**Fig. 1:**
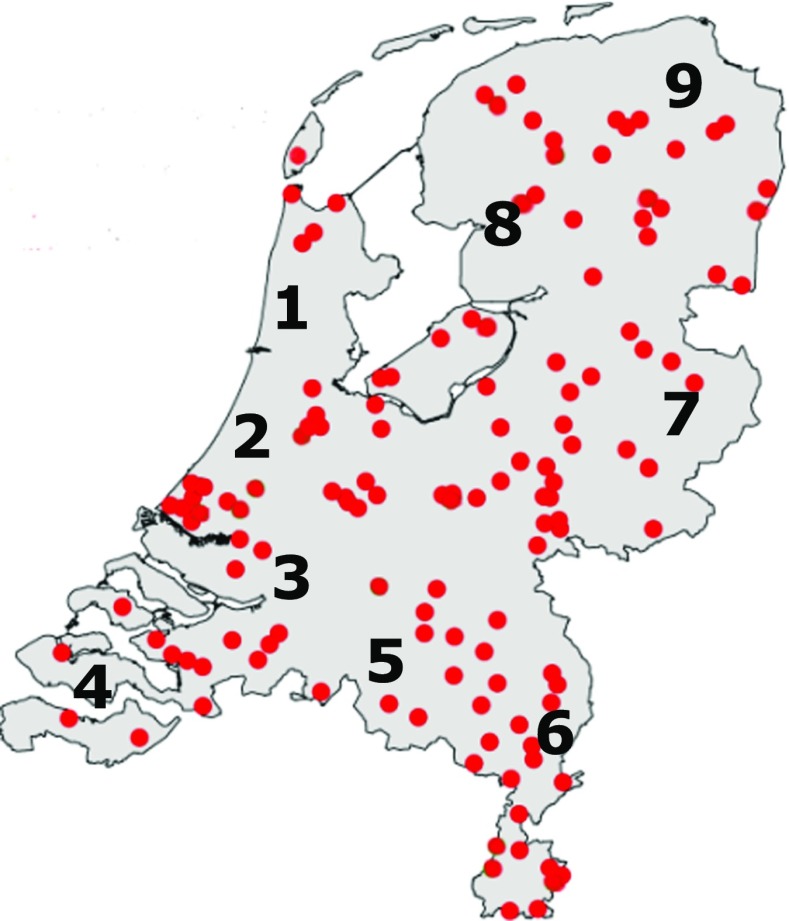
Location of the apiaries sampled. Numbers *1*–*9* indicate the postal codes zones 1–9

The geographic distribution of apiaries sampled is presented in Fig. 1.

### Statistics

Of the metals not detectable in an apiary sample, 1/2 limit of detection (LOD) value is set in the database. Per metal in the 150 apiary dataset, the median, lower quartile (25 % percentile), upper quartile (75 percentile), arithmetic mean, min/max and standard deviation (SD) were calculated.

The means and statistical differences of the metal concentrations per land use surrounding the apiary are calculated with analysis of variance at a *p* level of 0.05. Differences between means exceeding the least significant difference (LSD) are considered significantly statistically different. This dataset consisted of 147 apiaries used for bee disease check out of the 150 apiary dataset. Of multiple apiaries owned by one beekeeper, one apiary was included in the disease and land use analysis. The calculations were done with Genstat 12 Ed.

### Regions

The regions are based on the postal codes. In Table 1, per first postal code number and the corresponding part of the Netherlands are presented.

**Table 1:** General description of the Netherlands in postal code regions

Postal code	General localisation of the regions
1	Northern part of Noord Holland and Gooi
2	Southern part of Noord Holland and northern part of Zuid Holland
3	Southern part of Zuid Holland and Utrecht
4	Zeeland and western part of Noord Brabant
5	Mid- and eastern part of Noord Brabant and the northern part of Limburg
6	Mid- and southern part of Limburg and region Nijmegen/Arnhem
7	Gelderland minus region Nijmegen/Arnhem, eastern part of Overijssel and Drenthe
8	Western part of Overijssel and western part of Friesland
9	Eastern part of Friesland and Groningen

## Results

### Metals

The concentrations of metals per gram dry matter worker bee of pooled samples per apiary are presented in Table 2. The median and mean differ slightly, showing that the data are not completely normally distributed; they are skewed to the higher concentrations. Nevertheless, the normal distribution appeared to be the best-fitting distribution.

**Table 2:** Metals in honeybee workers (in micrograms per gram dry matter bee) of pooled samples of five colonies per apiary of 150 apiaries

Metals	Median	Lower quartile (25 percentile)	Upper quartile (75 percentile)	Mean	Min/max	SD
Al^a^	15.55	11.88	22.90	17.75	4.95/43.90	8.01
As^b^	0.85	0.56	1.03	0.79	0.13^c^/1.64	0.33
Ba	1.84	1.30	2.40	2.05	0.27/8.68	1.25
Cd	0.22	0.15	0.31	0.24	0.05/0.73	0.13
Co	0.16	0.14	0.22	0.19	0.08/0.63	0.08
Cr	0.39	0.33	0.52	0.45	0.19/1.42	0.19
Cu	19.25	17.2	22.5	20.00	11.70/32.2	4.13
Li^b^	0.01	0.01	0.03	0.03	0.01^c^/0.13	0.02
Mn	154	81.70	226.00	167.70	31.30/524.00	106.40
Mo	0.68	0.55	0.84	0.75	0.35/5.28	0.44
Ni	0.55	0.41	0.76	0.60	0.13/1.48	0.26
Sb^b^	0.30	0.13	0.43	0.31	0.13^c^/3.22	0.29
Se	1.96	1.46	2.56	2.10	0.77/4.37	0.81
Sn^b^	0.35	0.27	0.44	0.39	0.13^c^/3.30	0.34
Sr	1.80	1.33	2.15	1.82	0.66/4.59	0.69
Ti	0.42	0.30	0.58	0.48	0.10/2.99	0.32
V^b^	0.03	0.013	0.05	0.04	0.01^c^/0.32	0.04
Zn	95.75	83.50	114.00	100.4	56.60/170.00	22.65

^a^Al was analysed in 149 samples; one analysis failed

^b^Not detected in all apiaries. As, Li, Sb, Sn and V were not detected in respectively 7, 84, 62, 30 and 62 apiaries

^c^1/2 LOD

**Fig. 2:**
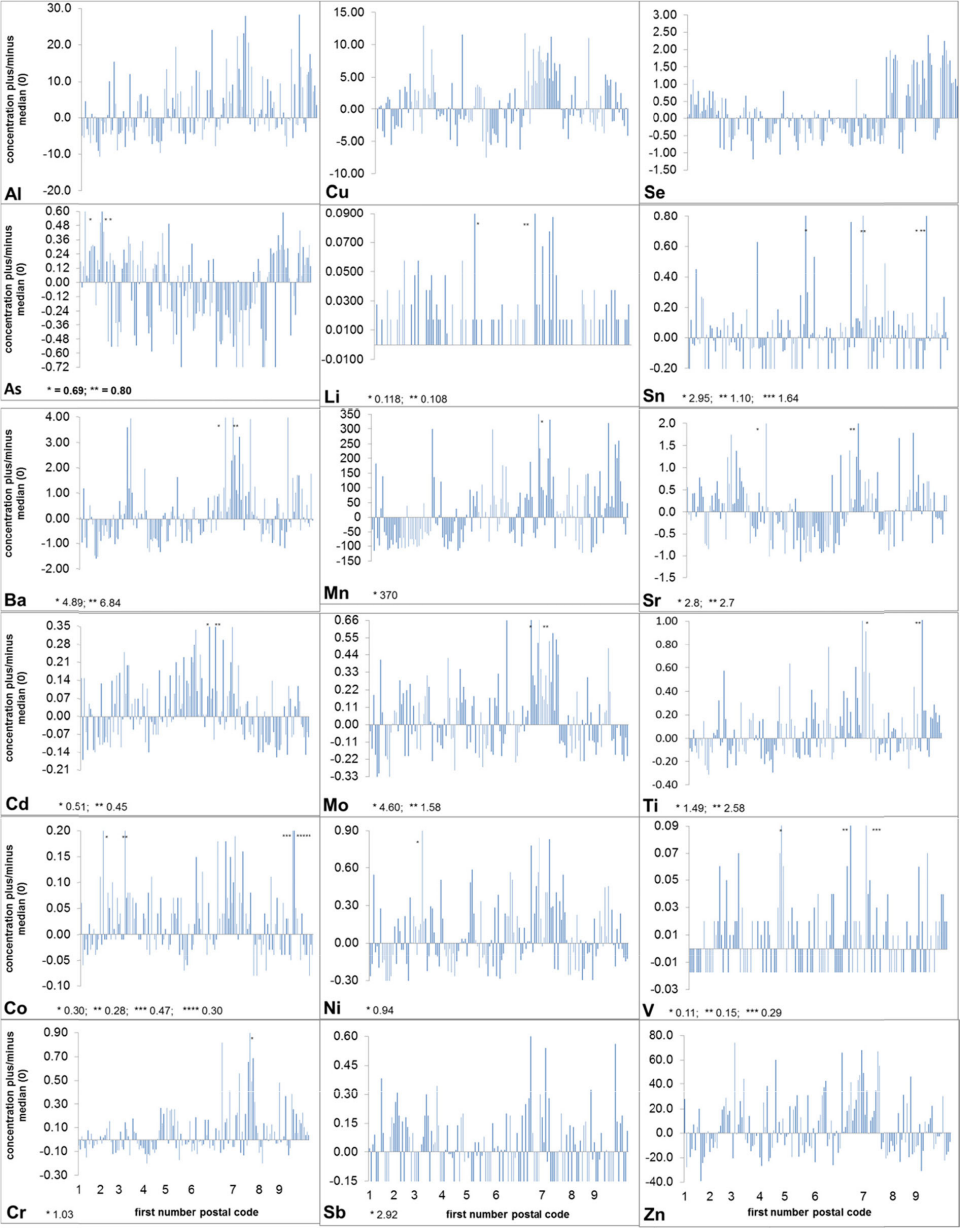
Concentrations of metals (in micrograms per gram dry matter bee) displayed as concentrations above and below the median (Table 2). The median is set on 0. The results per region of the postal codes are shown between the subsequent numbers of the first number of the postal codes (Table 1). The exact *even bars* in Li (plus), Sb (minus), Sn (minus) and V (minus) show that the analysis results are below the LOD and taken into the calculations as 1/2 LOD. Data exceeding the scale of the graphs are marked with an *asterisk*, and the values are displayed *next to the metal symbols* in the respective graph legends

In Fig. 2, the concentrations of the 18 metals and the 150 apiaries are presented as micrograms metal per gram dry matter bee above and below the median (concentration minus median). In Fig. 2, the median is set as 0. For reading the actual concentrations per apiary from Fig. 2, the median (Table 2) should be added.

### Land use

There is a significant difference in metal concentrations in bees in apiaries located at different land use sites. All metals except As, Se, Sn and V are recorded in significantly increased concentrations in >50 % wooded areas. Metal concentrations in >50 % agricultural areas, >50 % urban area and mixed land use show no significant difference (<LSD). The mean metal concentrations per land use are presented in Table 3.

**Table 3:** Metals (in micrograms per gram dry matter bee) per land use

Metals	>50 % agricultural area (*n* = 94)	>50 % woods (*n* = 7)	>50 % urban area (*n* = 16)	Mixed land use (*n* = 30)	LSD	*p*
Al	17.33	29.43	16.06	17.04	5.92	<0.001
As	0.79	0.53	0.93	0.80	0.25	0.066
Ba	1.84	4.52	2.02	2.24	0.88	<0.001
Cd	0.23	0.38	0.18	0.28	0.09	0.002
Co	0.18	0.26	0.19	0.18	0.06	0.041
Cr	0.45	0.66	0.38	0.42	0.14	0.007
Cu	19.58	26.40	20.01	19.46	3.03	<0.001
Li	0.02	0.06	0.04	0.02	0.02	<0.001
Mn	162.40	326.10	92.20	188.00	76.48	<0.001
Mo	0.70	1.71	0.68	0.70	0.30	<0.001
Ni	0.57	0.98	0.58	0.61	0.20	0.001
Sb	0.29	0.84	0.29	0.30	0.21	<0.001
Se	2.18	1.77	2.02	2.05	0.63	0.518
Sn	0.40	0.47	0.40	0.37	0.26	0.908
Sr	1.75	2.6	1.93	1.75	0.52	0.013
Ti	0.47	0.82	0.47	0.45	0.24	0.042
V	0.04	0.07	0.03	0.03	0.03	0.100
Zn	98.50	138.60	96.80	98.40	16.52	<0.001

## Discussion

### Honeybees

Forager bees are good samplers (Satta et al. 2012). In the current study, in-hive bees were sampled. In-hive bees taken from the outer frame of the brood box represent the average bee in the colony (Steen et al. 2012a). In a hive, particles on the bee’s body exchanged via physical contact (Degrandi-Hoffman et al. 1984; Free and Williams 1972; Paalhaar et al. 2008) and in the nectar via trophallaxis. Following the objective of the study—collect data on the spatial variation of metal concentrations in honeybee colonies in the Netherlands—sampling of in-hive bees and subsequent pooling of the bees per apiary were preferred over sampling of forager bees of individual hives per apiary. This study was a surveillance study to record spatial variation and not a bioindication study focussed on a possible explanation of differences of metal concentrations in bees.

### Metal concentrations

Based on the trend line (not shown in Fig. 2) that can be drawn in Fig. 2, Al, Ba, Cr, Mn, Mo and Se are present in higher concentrations in the eastern part of the Netherlands, whereas As shows the opposite. Cd, Co, Cu, Li, Ni, Sb, Sn, Ti, V and Zn show a horizontal trend line over the regions, showing no higher concentrations in bees in the east or west of the Netherlands. Figure 2 reveals regional patterns. Generally, per metal and per region, the concentrations above or below the median are clustered. Relatively high concentrations of Al, Ba, Cr, Cu, Mo, Ni and Zn are concentrated in the region roughly bordered by the cities Arnhem, Apeldoorn, Enschede and Winterswijk (northern part of postal code zones 6 and 7). Se shows two regions with high values: the region Zuid Holland (postal code zone 3) and the region Oost Overijssel, Drenthe, Friesland and Groningen (postal code zones 7–9). All apiaries are ranked in ascending postal code numbers up to the four numbers, and the bars in Fig. 2 represent apiaries in each other’s vicinity within the specific postal code region. More in detail, it can be seen that for all metals, in-region concentrations vary, showing a local effect. In praxis, this means, for heavy metal studies with honeybees: ‘think regionally, act locally’. Besides spatial variations, also temporal variations have been reported (Steen et al. 2012b). The current study has been performed once. Studying metal concentrations in bees in the Netherlands in another time of the year might give a different outcome.

As shown, metals are present in honeybees in a broad concentration range. It is the result of the actual presence of metals in the food (pollen, nectar, honeydew and water), biological presence of metals in a bee’s body, plus, possibly, metals deposited in the flowers from atmospheric deposition of metal-containing particles. The findings indicate significant differences in exposure ranging from low to zero up to high exposure.

In a previous study on the spatial and temporal variations of metal concentrations in adult honeybees (Steen et al. 2012b), concentrations exceeding significantly the mean (*p* ≤ 0.05) were considered to indicate a higher exposure. In bioindication studies by Porrini et al. (2002) and Gutiérrez et al. (2015), high (upper quartile 75 percentile) and low (lower quartile 25 percentile) reference thresholds (Table 3) are applied based on study results in Italy (Porrini et al.) and Spain (Gutiérrez et al.). In these studies, concentrations above the 75 percentile quartile were considered to be worrisome. The Ni, Cr and Cd data recorded in the current study are within the safe range, according to Porrini et al. (2002). Cr recorded in the current study would be worrisome, taking the high and low reference thresholds set by Gutiérrez et al. (2015).

As shown in Table 4, high and low reference values differ significantly per study, demonstrating the broad range of concentrations of heavy metals in honeybees. This variation is both temporal and spatial and therefore only applicable under defined conditions (Steen et al. 2012b). The current surveillance study implies only spatial variation as the samples were taken in a limited time window of about 1 week in June 2008. Compared to the mean concentrations of metals in micrograms metal per gram dry matter honeybee in the study of Steen et al. 2012b conducted in 2006 at three locations, the metals Al, Cr, Mn, Ni, Sb, Se, Ti and Zn show higher mean concentrations, but are all, except Cr and Mn, within the 95 % probability area of metal concentrations in the current study (mean + 1.66 × SD, one-sided). Compared to previously reported reference data (Steen et al. 2012b), the mean concentrations of As, Cd, Cr, Cu, Mn, Ni, Se and Zn recorded in the current study are all, except Mn and Ni, in the same range as detected in reported control sites (Bromenshenk et al. 1985; Velemínský et al. 1990; Fakhimzadeh and Lodenius 2000; Porrini et al. 2002; Roman 2005). The Mn and Ni concentrations exceeded the reported concentrations (Kump et al. 1996; Porrini et al. 2002; Roman 2005). The concentration ranges published in the current study show, for each metal, a large variation. Based on the demonstrated regional differences in the current study, threshold limits should be set per region and land use should be taken into account (see “Land use”). Hives in the same apiary show different metal concentrations in the bees (Steen et al. 2012b). Sampling multiple colonies per apiary provides a better overview of foraging sites within the foraging area of an apiary. Bees of colonies in one apiary divide themselves of the foraging area (Waddington et al. 1994). Therefore, pooled apiary samples can do for this type of surveillance study.

**Table 4:** High and low reference thresholds (in milligrams per kilogram wet matter bee)

	Porrini et al. (2002)	Gutiérrez et al. (2015)	Current study^a^
Pb	0.40–2.0	0.3–0.7	
Ni	0.10–0.40	0.1–0.3	0.13–0.24
Cr	0.04–0.25	0.04–0.12	0.11–0.17
Cd		0.052–0.1	0.05–0.10

^a^The data of the current study presented in Table 2 in dry matter bee are converted to wet matter bee, taking into account the weight loss of the drying process of 68 % (Steen et al. 2012b)

### Land use

The selection of the apiaries was not directed by land use but by the requirement of an overall coverage of apiaries over the Netherlands. As shown in Table 3, the majority of the apiaries sampled are in agricultural areas, next in mixed land use areas, then in urban areas and at the rear-end wooded areas. Despite the low numbers of apiaries in >50 % wooded areas, statistically significantly higher concentrations were recorded there, indicating the impact of land use on metal concentrations in bees. This phenomenon was also observed by Lambert et al. (2012). Further studies on the impact of land use should be done to reveal the mechanisms. Non-comprehensive reflections on why bees in wooded areas have higher concentrations of metals are that, at wooded sites, atmospheric deposition is greater in a forest interior than in a forest edge (Fowler et al. 2004). This may be due to decreasing wind speed in wooded areas (Raynor et al. 1974; Pleijel et al. 1996). Additionally, trees promote vertical transport by enhancing turbulence (McDonald et al. 2007). Honeydew resulting in sticky leaves is assumed to be more prevalent in wooded areas than in others, possibly resulting in an exceeded physical binding of metal-containing particles from atmospheric deposition (Dr. R. Moosbeckhofer, personal communication). Relatively low pH values in wooded areas increase the bioavailability of metals (Hutchinson and Whitby 1977; Takáč et al. 2009; Messenger 1986). This may also contribute to the increased exposure of metals to honeybees. In general, dark honeys contain honeydew and have higher metal concentrations compared to light honey (Crane 1979). These typical features of a wooded site may affect increased deposition of airborne metal-containing particles originating from other locations.

Measuring the metal concentrations in honeybees for bioindication purposes is an indirect recording of the sum of metals in pollen, nectar and honeydew, plus, possibly, additional deposition. This sum cannot be split in the two terms as deposition of heavy metals is not recorded separately. This is the intrinsic uncertainty of heavy metal bioindication studies with honeybee colonies. Elevated concentrations of specific metals are always the result of a higher exposure, but this needs not to be one-to-one related to the level of deposition.

## Conclusion

Honeybee colonies proved to be applicable as bioindicators of metal burden in the regional and local environments. Honeybees in apiaries in different regions in the Netherlands have different concentrations of metals, a specific regional effect. Within the regions are local differences. The data indicate higher metal concentrations in >50 % wooded areas compared to >50 % agricultural, >50 % urban and mixed used areas, a local effect.

For qualitative bioindication studies, regional and local effects should be taken into account. Furthermore, land use effect should be studied in detail to reveal the mechanisms resulting in different concentrations of metals in bees. Both regional and local effects have consequences for conclusions on overexposure of bees to metals in comparison studies.
